# The effectiveness of an adapted modular CBT training for youth with depressive symptoms: a study protocol for an n-of-1 trial

**DOI:** 10.1186/s40359-026-04252-8

**Published:** 2026-03-07

**Authors:** Jennifer de Lange, Liesbeth de Paauw-Telman, Marieke van den Heuvel, Anouk Vroegindeweij, Denise Bodden

**Affiliations:** 1 Utrecht University, Utrecht, Netherlands; 2 Ede Christian University of Applied Sciences, Ede, Netherlands; 3 University Medical Center Utrecht, Utrecht, Netherlands

**Keywords:** Youth, Depression, Prevention, CBT, Single case study

## Abstract

**Background:**

The prevalence of depressive symptoms among youth seems to be increasing. Depression among youth is associated with poorer academic performance and social relationships. Preventive interventions for youth with elevated depressive symptoms are essential. The STARr training was found to reduce depressive symptoms in youth; however, it was too linguistic and could be enhanced in terms of gender and cultural background. This study will examine whether a simplified, adapted modular CBT training (STARr 2.0) is effective in reducing depressive symptoms and co-occurring anxiety symptoms among youth with depressive symptoms. Further, this study will assess whether the module sequence preferences of adolescents, trainers, or data predict the outcome depressive symptoms. Additionally, alliance, motivation, and expectation of the treatment will be assessed as predictors. Last, we will examine moderators (age, gender) and change mechanisms (problem solving, cognitive restructuring, behavioral activation, and stress).

**Methods:**

An n-of-1 study with a single-case observational design with a baseline and effect phase will be employed. The baseline phase starts three weeks before the training. We aim to include 30 adolescents between 12 and 20 years at two mental health organizations. All participants receive the adapted STARr-training in groups of six to eight participants. The training consists of 12 hourly sessions provided by a trained trainer. Participants receive in total three short questionnaires per week and six longer questionnaires (start baseline, before the start of the training, and after each module). The primary outcome is depressive symptoms and secondary outcomes are anxiety, stress, satisfaction with the training, and individual and societal functioning.

**Discussion:**

The findings of this study will provide insight into whether an adapted simplified version of the STARr training is effective in reducing depressive symptoms and will also provide insight into change mechanisms and potential subgroup differences. If the adapted version is effective in reducing depressive symptoms, then the training can be used widely by mental health professionals.

**Trail registration:**

Overview of Medical Research in the Netherlands (*OMON).* Registration number NL-OMON58273, registration date: 4 December 2025.

## Background

### Depression and cognitive behavioral therapy

Depression is highly prevalent among youth [1, 2]. Meta-analytic studies show that within Europe, 1,7% of children and adolescents suffer from a depressive disorder [2], and around 14% of youth worldwide suffer from subclinical depression [3]. Also, in the Netherlands, the prevalence of mental health problems seems to have increased between 2017 and 2022 [4, 5]. Depression in youth often co-occurs with anxiety and stress symptoms [6, 7], and is associated with severe negative consequences such as poorer academic performance [8, 9], poorer social relationships [10], and suicidal ideation [11]. Moreover, youth depression is associated with unemployment, depression, anxiety, and suicidality in adulthood [12, 13]. Societal impact of depression is also high, as societal costs amount to 38 million euro annually for clinically depressed youth [14] and 42 million euro annually for subclinical depressed youth [15]. Given the increase in prevalence in mental health problems and the detrimental consequences of untreated depression, preventive interventions for youth with elevated depressive symptoms are essential.

Cognitive behavioral therapy (CBT) is the first choice of psychotherapy and has proven to be effective in reducing subclinical levels of depressive symptoms in adolescents [16–18]. However, not all adolescents with depressive symptoms seem to benefit from receiving CBT [17, 19]. Therefore, it is essential to assess the requirements for adapting CBT for adolescents. In response to the need to improve therapy outcomes, clinicians and researchers have moved away from the one-size-fits-all approach for CBT and have developed personalized interventions for youth [20]. A meta-analysis showed that personalized interventions seem to improve psychological therapy outcomes compared to standardized therapy [21]. Over the last few decades, evidence has been found for methods of both tailoring treatment to the individual based on personal characteristics, as well as adapting the treatment during the therapy process [20]. These personalized interventions include modular therapy, shared decision making, individualized metrics, and adaptations regarding inclusivity and diversity [20, 22].

One method to optimize and personalize treatments for youth is a modular approach. In a modular approach, the sequence of CBT modules can be adjusted, omitted, or repeated based on the client’s needs, preferences, and characteristics [20, 23]. Several studies found that modular psychotherapy (e.g., CBT) was associated with a decrease in depressive or anxiety symptoms in young people [24–26]. Moreover, modular psychotherapy was associated with better treatment outcomes than non-modular psychotherapy among youth with depressive or anxiety symptoms [27].

Our previous study examined a modular preventative intervention, based on CBT, for youth aged between 10 and 20 years old with elevated depressive symptoms. This intervention is called STARr (Solve (problem solving), Think (cognitive restructuring), Act (behavioral activation), Relax (relaxation), and repeat) [28]. For further details of this study, see van den Heuvel et al. [26, 28, 29]. A randomized controlled microtrial was conducted, including 282 participants between the ages of 11 and 18 years old (*M* = 13.82, *SD* = 1.48) with elevated levels of depression. It was found that STARr significantly reduced depressive symptoms when adolescents received all four modules [26]. The distinct modules did not significantly reduce depressive symptoms (after three sessions), and the reduction of depressive symptoms was not affected by the sequence of the modules [26]. The reduction of depressive symptoms was also not moderated by gender, age group, or severity of depressive symptoms [29]. This suggests that modular CBT is promising in reducing depressive symptoms in a wide range of youth.

A second way to further optimize and personalize CBT is the use of shared decision-making in therapy [30]. Shared decision-making is a process in which the clinician and client discuss treatment decisions and plans and reach consensus [31]. Shared decision-making can be used for deciding the sequence of modules in a modular approach, such as the STARr training. Systematic reviews on shared decision making and patient preference found little evidence that shared decision-making improves mental health outcomes (e.g., depression, quality of life, anxiety) [30, 32–34], but found an increase in treatment adherence and satisfaction [30, 33, 35]. Most of these studies have included adult samples. Further studies are needed to examine whether shared decision-making improves mental health outcomes in youth, preferably including an assessment of fidelity and a clear description of decision-making methods [30, 32].

Shared decision-making is based on the clients’ and therapists’ perspectives; however, decision-making can also be informed by data. Statistical (data-driven) models or algorithms (the Personalized Advantage Index: PAI) can guide the decision-making regarding the sequence of treatment elements or what treatment would likely be best for the client [22, 36]. Based on several characteristics of a client, the PAI can predict what sequences of modules would most likely lead to the most optimal treatment outcome for an individual [22]. This method could inform therapists in deciding on which module to use. Previous studies among adults have examined differences in outcomes predicted by the PAI and found that participants who were assigned to the predicted optimal treatment had better treatment outcomes (e.g., lower predicted depression scores) than those who were not [22, 37–39]. Nevertheless, a systematic review on decision-making in modular treatment for youth showed that none of the treatments utilized statistical models or algorithms to make decisions in treatment [40]. This indicates that evidence of data-driven decision making is missing for youth, and it is necessary to assess whether algorithms can aid in predicting the optimal module sequence in modular treatments for youth. Furthermore, we want to explore whether other treatment characteristics (besides preference) predict the reduction in depressive symptoms; these predictors are motivation, expectancy, and alliance.

A third method to optimize and personalize interventions is by making them more inclusive. Evidence suggests that when CBT is inclusive of diverse educational levels, cultural backgrounds, and gender, alliance and satisfaction are rated higher by participants [41–44].

Both alliance and satisfaction are associated with better clinical treatment outcomes [45, 46].

Evaluation of the STARr intervention revealed that not all youth seemed to benefit at an individual level [26]. Focusgroups with youth and trainers showed, among others, that the training and booklets were too linguistic, and that the sessions could be more active (de Lange et al., in preparation). Additionally, the interventions’ inclusivity could be improved regarding cultural background and sexual and gender identity. Thus, to further optimize and personalize the STARr intervention, the intervention was adapted and simplified (de Lange et al., in preparation).

Last, examining for whom and how CBT works could also aid in improving treatment [47, 48]. A systematic review on moderators of treatment outcomes of psychological interventions found only one study that indicated that CBT had better treatment outcomes for girls compared to care as usual, but other studies did not find moderation effects for gender [49]. This is in line with van den Heuvel and colleagues’ study [29], which did not find differences for gender when comparing behavioral and cognitive modules. Regarding adolescents’ age, no moderation effect was found in studies in the systematic review [49], and no differences for age were found when comparing behavioral and cognitive modules in van den Heuvel and colleagues’ study [29]. In the current study, we will examine whether certain module sequences lead to better treatment outcomes depending on gender and age.

With respect to mechanisms of change in psychotherapy, a meta-analysis on CBT for depression in adults found small-to-medium effect sizes for cognitive processes (e.g., changes in negative cognitions) and small effect sizes for behavioral strategies (e.g., changes in activation) were observed [50]. Huibers and colleagues [48] suggest that besides cognitive and behavioral mechanisms of change, other processes might act as a mechanism of change, such as therapeutic alliance. Thus, it is important to assess mechanisms of change (e.g., cognitive restructuring and behavioral activation) for the STARr training.

In short, optimizing and personalizing CBT interventions is necessary to promote adherence and motivation. Several possibilities exist to optimize and personalize cognitive behavioral therapy for youth with depressive symptoms. For example, utilizing a modular approach, utilizing shared decision making in therapy, making interventions more inclusive, and lastly gaining insight in what works for whom.

### Current study and objectives

The current study is a follow-up study of the “STARr-project: Preventing depression in youth” [28], which was conducted between 2017 and 2020.

The current study will investigate:

1. whether a simplified, adapted and more personalized version of the modular STARr-training (STARr2.0) is effective in reducing depressive symptoms (and comorbid stress and anxiety symptoms) among adolescents aged between 12 and 20 years. The effect size of the reduction in depressive symptoms will be compared with the effect size from the previous STARr study. Additionally, treatment satisfaction and drop-out will be assessed as secondary outcomes. Additionally, the descriptive statistics of satisfaction and drop-out will be compared to the previous STARr study.
2. whether the preference of the adolescents, the preference of the trainer, or a data-driven model regarding the sequence of modules best predicts a reduction in depressive symptoms (and comorbid stress and anxiety symptoms). And whether treatment characteristics (motivation, expectancy, and alliance) predict a reduction in depressive symptoms.
3. for whom and under which circumstances (moderators; gender and age) the modules of the STARr training works best and
4. which mechanisms of change drive the reduction in depressive symptoms (mediators; problem solving, negative thinking errors, behavioral activation, and stress).

To assess the adapted modular approach (STARr2.0), a single case observational design (SCOD) will be utilized [51]. A single case design is highly feasible for clinical studies, because it allows for evaluating the efficacy of individual trajectories, and thus small samples [52, 53]. We will use an AB-design with a non-randomized introduction of the intervention.

## Methods

### Ethics

Approval was obtained from the ethics committee of the Faculty of Social & Behavioural Sciences of Utrecht University on 26 September 2025. The ethics committee will be informed of all significant study amendments. The study is registered at the Overview of Medical Research in the Netherlands [54] (NL-OMON58273) on 4 December 2025. Results will be reported using SPENT guidelines [55].

### Study design

A single case observational design (SCOD) will be used to examine treatment effects [51]. More specifically, an A/B-phase design is used. Within this design, phases A and B may respectively represent the baseline and intervention phases – or all measurements before and after the expected start of the treatment effect (in case of interventions with delayed or lagged treatment effects) [51]. In our study, we expect that the treatment effect will start after the first module (i.e., three weeks after the start of the intervention). Therefore, we will label all observations collected before and during the first module as part of Phase A, and all observations after the first module as part of Phase B. This means that Phase A consists of 6 weeks (3 weeks baseline plus 3 weeks module 1) and Phase B consists of 9 weeks (modules 2, 3, and 4). The outcome variables of interest will be monitored through repeated measurements three times per week in both phases [51]. Although the current study design does entail an experimental intervention, we refer to the design as ‘observational’ because the start of the intervention cannot be randomized across participants – a procedure that usually increases the internal validity of the single case design [56]. The start cannot be randomized for practical reasons at the institutes’ organizational level and for ethical reasons (e.g., potentially postponing the start of training for some participants). In such cases, it has been suggested to observe the start of the intervention as a ‘natural event’ and analyze the data with statistical methods that treat the data accordingly [51]. All participants in one group will start the training at the same time, three weeks after completing the baseline questionnaire. The study ends with a follow-up questionnaire after the last training session has ended. There is no additional follow-up phase with repeated measurements incorporated.

### Participants and recruitment

In total, 30 participants will be included in the study. Youth who seek help for depressive symptoms, anxiety symptoms and/or stress that are advised to follow the STARr training are eligible to participate. To be eligible to participate in this study, a participant needs to be registered for the STARr training at one of the two participating mental health organizations and be between 12 and 20 years old. When a participant has acute and severe suicidal thoughts and/or intentions (a score of 2 on the Children’s Depression Inventory-2 (CDI-2) item ‘a desire to kill oneself, if given the chance’ on the CDI-2 [57] in combination with a total score of 12 or higher on the suicide items of the Questionnaire about suicide and self-harm [58], the trainer will be notified. The trainer will follow the organization’s protocol regarding suicidality and will decide whether the participant can continue the training.

Participants will be recruited through the two mental health organizations. Youth who register at one of the two organizations for a STARr intervention will receive information about the study and will be invited to participate. Youth will receive an information letter and the informed consent form. If youth are aged between 12 and 16 years old, their parents will also receive an information letter and an informed consent form. Youth have two weeks to decide whether they want to participate, and they can provide informed consent online through Qualtrics. Parents of youth aged between 12 and 16 years will also have to provide consent for their child. If youth do not want to participate, they will still receive the STARr training. They can also withdraw from the study at any time without explanation and continue the STARr training. When a participant withdraws from the STARr training and the study, they will receive an exit questionnaire that includes the same items as the long questionnaires along with an additional open-ended question inviting them to elaborate on their reasons for discontinuing the STARr training.

### Sample size and power calculation

We used a power calculation application for single-case designs [59] to calculate the expected power when utilizing a permutation distancing test with a total of 30 participants who complete the study. Participants will receive a total of 45 repeated measurements, of which 18 belong to Phase A and 27 to Phase B. With a mean difference of 1.5 units (small treatment effect) between phases A and B (SD phase A = 3 and SD phase B = 3), and a missing percentage of 20% and autocorrelation level of 0.2, the power would be 79%. It is important to note that this is the calculated overall power is calculated for the group. The power per individual could be lower or higher, depending on, e.g. the percentage of missing observations and the level of autocorrelation between observations.

### Intervention

STARr is a group training and takes place in groups of six to eight participants. The STARr-intervention consists of four modules that are based on the core elements of CBT for depression: problem solving (Solve), cognitive restructuring (Think), behavioral activation (Act&do), and relaxation (Relax). These elements are theoretically well-founded and incorporated in most CBT interventions for young people with depressive symptoms [17, 60, 61]. Participants receive a combination of these four modules in weekly sessions (3 sessions per module), and the program’s total duration is 12 weeks [28]. Participants receive a workbook that contains (homework) exercises for each module.

The adapted version of the STARr training (STARr2.0) is grounded in the same theoretical framework as the original STARr training. Accordingly, it remains a modular, preventative group training consisting of 12 one-hour sessions targeting youth aged 12 to 20 experiencing depressive symptoms and/or (comorbid) stress and anxiety. To enhance accessibility and inclusivity, several adaptations were made: workbook texts were shortened and rewritten at B1 level, images and more active exercises were added to support understanding, and more diverse examples reflecting educational, cultural, sexual, and gender diversity were included. The adapted training that will be investigated will be delivered in groups of six to eight participants.

### Procedure

**Fig. 1:**
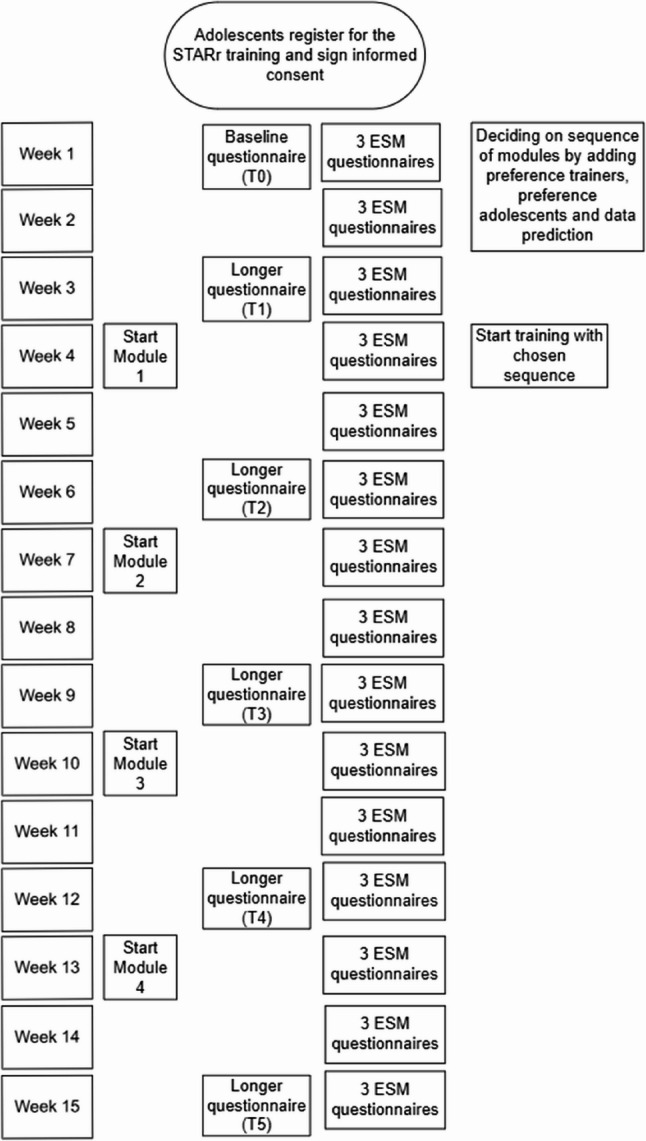
Procedure and data collection points

The STARr training will be provided by two mental health organizations in the Netherlands. Professionals from both organizations were trained by two researchers/psychologists from the research team who developed the STARr training. The trained professionals will deliver the training. See Fig. 1 for an overview of the procedure and data collection points.

Participants will receive one of the four module sequences assessed in the previous STARr study, which has been shown to be associated with a significant reduction in depressive symptoms. The following sequences of modules were assessed in the previous STARr study: Think-Act-Relax-Solve (1), Act-Think-Relax-Solve (2), Solve-Act-Think-Relax (3), and Relax-Solve-Act-Think (4). The sequence will be based on three factors: youth’s preference, trainer’s preference, and a data-driven prediction. During the intake, the trainer explains the content of the four modules to the participant and asks the adolescent which sequence of modules (options 1, 2, 3, or 4) would fit best based on the participant’s needs and struggles (participant’s preference). Particular attention is given to the first preferred module (problem solving, cognitive restructuring, activation or relaxation). Next, trainers indicate to the researcher their preferred module order for each participant, based on the intake and the nature of the participant’s needs and symptoms (trainers’ preference). Finally, an algorithm determines the most suitable module sequence per participant (PAI) [22] (data-driven prediction). This data-driven prediction uses an algorithm that is based on data from the previous STARr study [26]. Personalized Advantage Indexes (PAI) were calculated using a random forest algorithm [22, 62]. Using the random forest algorithm, the PAI-scores are calculated for participants of the current study using the baseline questionnaire data. The variables included in the algorithm are gender (boy/girl), age, Dutch/non-Dutch ethnicity, and variables regarding depressive symptoms, comorbidity, problem-solving approaches, negative cognitive errors, behavioral activation, and stress.

The two preferences (participant and trainer) and the prediction (data-driven) of all participants in one training group are then summed up, and the most frequently selected sequence is delivered in that group. These three preferences are subsequently used as predictors for the study outcomes.

### Measures

The following measures are included in the study. Table 1 provides an overview of the timing of each measurement.

**Table 1:** Overview of assessments

Type of variable	Domain/ Concept	Instrument		Assessment					
			Items	A	T	t_0_	T_w_	T_m_	T_*p*_
Primary outcome	Depressive symptoms	CDI-2	28	x		x		x	x
		CDI-2 2 item	2				x		
Secondary outcome	Depressive symptoms	NRS for core symptoms of depression	3	x		x		x	x
	Anxiety symptoms	NRS 2 items	2				x		
		SCARED-5 + items social anxiety	13	x		x		x	x
		SCARED 2 item	2	x			x		
	Top three problems	TP measure	3	x		x			x
	Suicidal ideation (if score 2 on item 8 CDI-2)	VOZZ suicide items	8/4	x		x		x	x
	Comorbidity	BPM	19	x		x		x	x
	Individual societal impact questionnaire	ISIQ	10	x		x		x	x
Mediators	Negative cognitive errors	CNCEQ-R	16	x		x		x	x
		CNCEQ-r 2 items	2				x		
	Behavioural activation	BADS	25	x		x		x	x
		BADS 2-item	2				x		
	Problem solving skills	SPSI-R	10	x		x		x	x
		SPSI-R 2 items	2				x		
	Relaxation	PSS-10	10	x		x		x	x
		PSS-10 2 items	2				x		
		NRS for relaxation	1	x		x		x	x
Moderators	Demographics adolescent			x		x			
Treatment characteristics	Current and previous treatment	VEHI	6	x		x			x
	Expectancy of treatment	PETS	7	x		x			
	Therapeutic alliance	TASC-r	12	x				x	x
	Satisfaction treatment	SSS	3	x				x	
	Youth’s motivation for treatment	MYTS	8	x		x		x	x
	Module questionnaire		5	x				x	x
	Drop-out / amount completed sessions				x				
	Treatment integrity				x			x	

*Abbreviations*: *A* adolescent, *FV* full-length version, *NRS* Numerical Rating Scale, *SV* short version, *T* therapist, *t*_*0*_ baseline assessment, *t*_*w*_weekly assessments (three times a week), *t*_*m*_ post-module assessment, *t*_*p*_ post-intervention assessment

*Depressive symptoms* are assessed using the Children’s Depression Inventory-2 (CDI-2) [57]. The CDI-2 consists of 28 items, and response options are rated on a 3-point scale. A higher score indicates more severe symptoms. The CDI-2 has demonstrated good reliability and validity [57]. The Cronbach’s alpha in the previous STARr study varied between 0.86 and 0.90 [26].

*Anxiety* is assessed with the Screen for Child Anxiety Related Emotional Disorders (SCARED)-5 [63, 64] and eight additional items from the social anxiety subscale of the SCARED-71 questionnaire [65]. These 13 items are rated on a 3-point scale ranging from “not true or hardly ever true” to “very true or often true”. The SCARED has demonstrated good reliability and validity [65].

*Individual and societal functioning* is assessed with the Individual and Societal Impact Questionnaire (ISIQ) [66]. The questionnaire includes questions about physical health, mental health, personal hygiene, school and work, friendships, romantic relationships, family, daily activities, independence and leisure time. Items are rated on a 6-point Likert scale ranging from “none” to “a lot”. The ISIQ showed good reliability [67].

*Suicide risk* will be assessed with eight items of the suicide and self-harm questionnaire (VOZZ) [58]. These items measure past and present suicidality. Response options are rated on a 5-point scale. A higher score indicates more severe symptoms. The VOZZ has demonstrated good reliability and validity [58].

*Comorbidity* is assessed with the Brief Problem Monitor (BPM) [68].

The BPM consists of 18 items divided into three subscales: internalizing, externalizing and attention problems. Response options are rated on a 3-point scale from “not true” to “very true”. The BPM has demonstrated good reliability and validity [68].

*Problem-solving approaches* in daily life will be assessed with the short form of The Social Problem Solving Inventory-Revised (SPSI-R) [69]. This measure consists of 10 items and includes five subscales: positive problem orientation, negative problem orientation, rational problem solving, impulsivity/carelessness style, and avoidance style. Response options are rated on a 5-point Likert scale ranging from “not at all true” to “extremely true of me”. The SPSI-R showed good reliability and validity [70].

*Negative cognitive errors* will be assessed with the Children’s Negative Cognitive Errors Questionnaire – Revised (CNCEQ-R) [71]. This questionnaire measures five negative cognitive errors: underestimation of the ability to cope, personalizing without mind-reading, mind-reading, selective abstraction, and overgeneralizing. Response options are rated on a 5-point Likert scale ranging from “not at all like I would think” to “almost exactly like I would think”. The CNCEQ-R showed moderate to good reliability and validity [71, 72].

*Behavioral activation* will be assessed with the Behavioral Activation for Depression Scale (BADS) [73]. The BADS consists of four subscales: activation, avoidance/rumination, work/school impairment, and social impairment. Response options are rated on a 7-point Likert scale ranging from “not at all” to “completely”. The BADS demonstrated acceptable to good reliability and validity [73–75].

*Stress* will be assessed with The Perceived Stress Scale (PSS-10) [76, 77]. Response options are rated on a 5-point scale ranging from “never” to “very often”. The PSS-10 has demonstrated acceptable reliability and validity [78].

#### Treatment characteristics

*Motivation for treatment* will be assessed by the Motivation for Youth’s Treatment Scale (MYTS) [79]. Eight items are rated on a 5-point Likert scale ranging from “strongly disagree” to “strongly agree”. The scale consists of two subscales: “recognition that the youth have a problem” and “readiness to participate in the youth’s treatment”. The MYTS is a psychometrically stable measure [79].

*Adolescents’ expectancy of each module* will be assessed with the Parent Expectancies for Therapy Scale (PETS) [80]. The measure was revised for adolescents [81], and we adapted the wording of the items so that it applies to each module of the training. Seven items are rated on a 6-point Likert scale ranging from “totally disagree” to “totally agree”. The original measure showed good reliability and validity [82].

*Therapeutic alliance* will be assessed with the Therapy Alliance Scale for Children-revised (TASC-r) [83]. The TASC-r consists of 12 items and response options were assessed on a 4-point scale ranging from “not at all” to “very much”. The TASC-r scores have demonstrated good reliability and validity [84].

*Satisfaction with the training and modules* will be assessed with the Service Satisfaction Scale (SSS) [85]. The scale consists of four items, and response options are rated on a 4-point scale ranging from “no, definitely not” to “yes, definitely”. The measure has demonstrated good psychometric properties [85].

*Demographic characteristics* include gender, age, ethnicity, and education level.

*ESM-questionnaire.* Experience sampling method (ESM) will be utilized to collect data. The ESM questionnaire consists of 14 items that are rated three times a week. It includes two items from the following measures: CDI-2, BADS, PSS-10, CNCEQ-R, SPSI-R, and SCARED. The items were selected based on which item loaded highest in a factor analysis using the data from the previous STARr study [26]. Regarding the CNCEQ-R, we changed the statements into the underlying negative cognitions ‘feelings of not being good enough’ and ‘feeling like a burden to others’. Additionally, two items about the core criteria of the DSM-5 major depressive disorder are included, namely loss of pleasure and low mood [86]. Participants receive the questionnaire on three days of the week between 8 a.m. and 8 p.m. One reminder is sent after 24 h, and participants are given 48 h to complete the questionnaire.

*Treatment integrity* will be assessed using a questionnaire completed by the trainer. Treatment integrity will be administered after each module and will include adherence, treatment differentiation, and child involvement [87, 88]. The researchers developed this questionnaire, which includes questions about practical elements (e.g., duration, pace, structure, participant involvement), content elements (e.g., psychoeducation, exercises, usage of worksheets, homework) and whether the specific goals per session are achieved.

### Data collection and management

Participants are asked to complete three short ESM questionnaires each week for 15 weeks, including the weeks before the start of the training (i.e., a total of 45 ESM questionnaires). These ESM questionnaires take around 1 min to complete.

Next to that, participants are asked to complete six longer questionnaires. The longer questionnaires are administered three weeks before the start of the training (T0), just before the start of the training (T1), and after each module (T2 -T5). These longer questionnaires take between 30 and 45 min to complete. Last, trainers are asked to complete questionnaires about treatment integrity after each session.

Data will be collected using m-Path [89, 90] and Qualtrics [91]. M-Path is a mobile application designed for clinicians and researchers to distribute daily questionnaires. Participants will receive a notification on their mobile phones to complete the questionnaire. Participants receive 7,50 euro compensation for each longer Qualtrics questionnaire and 1 euro for each ESM questionnaire they complete. Participants can receive up to 90 euros in compensation in total.

All data will be collected according to the General Data Protection Regulation (GDPR) [92]. Qualtrics and m-Path both comply with the GDPR [89, 91]. All participants receive a participant ID in Qualtrics and m-Path. This participant ID is used to connect the data from all data points. Participants’ identifiers, consent forms (which include email address and firstname) and the key will be stored in a separate folder with a password. Two researchers from the research team have access to this key. All data will be stored on a data management system provided by the institution (Utrecht University). This system is safe to use for privacy-sensitive data. When data collection is finished, consent forms, email addresses, and firstnames of participants will be deleted. All researchers from the research team have access to the deidentified dataset.

### Statistical analyses

#### Primary and secondary outcomes

The Permutation Distancing Test (PDT) [51] will be utilized to assess mean level differences of the primary outcome (*depressive symptoms)* between the baseline phase and the effect phase per participant. For this purpose, we will use the repeated measurements data (i.e., the 3 ESM measurements per week). The PDT was developed to evaluate individual treatment effects in SCODs. It is a nonparametric permutation test that accounts for autocorrelation by applying stepwise down-sampling while using all available observations to create sufficient ‘distance’ between observations (for more information, please see Vroegindeweij et al. [51]. In the PDT, the moment (the lag) is defined at which a treatment effect is expected to start. This marks the beginning of Phase B (i.e., the effect phase). The observations before Phase B are part of Phase A (i.e., the baseline phase) [51]. We expect that Phase B will start after the first module (three weeks after the start of the training). Hence, we will use a lag of 3 in our analyses for all participants. The PDT analyses will be performed with *R*-package *“pdt”* in Rstudio. The *R*-package includes a function to plot the individual time series to inspect the data for (linear) trends first. We shall use this function before performing the PDT analyses.

In addition, mean level differences of the secondary outcomes (*stress*,* anxiety*, and *individual and societal functioning)* will be assessed. Further, the descriptive statistics of the secondary outcomes, *satisfaction with the training and drop-out*, will be provided and compared with *satisfaction with the training* results from the previous STARr study [26].

Last, the youth’s, trainers’, and data-driven preferences of module sequences, and treatment characteristics (*motivation for treatment*,* therapeutic alliance*,* and participants’ expectancy of each module)* will be assessed as predictors for change in depressive symptoms. The mean level differences and predictions will also be performed in RStudio.

#### Mediation and moderation analyses

Next to analyzing mean level differences, *problem-solving*, *negative cognitive errors*, *behavioral activation*, and *stress* are assessed as mediators in the relation between depressive symptoms at T1 and T5. Assessing these as mediators will inform what mechanisms contribute to changes in depressive symptoms [93]. In addition, *gender* and *age* will be assessed as moderators. We will assess whether the interaction between these moderators and module sequence leads to different outcomes. We are only able to assess the moderators when various module sequences are executed during the study. Mediation and moderation analyses will be conducted in RStudio using multilevel regression modeling techniques [93–95].

#### Missing data

Depending on the amount of missing data and whether it is missing completely at random or missing at random, we will select an adequate imputation method.

#### Visualization

Additionally, the primary outcome (*depressive symptoms)*, and the mediators (*problem solving*, *negative cognitive errors*, *behavioral activation*, and *stress)* will be presented graphically. Graphically presenting these results might demonstrate patterns and trends during each module and the full training [96, 97].

### Dissemination

After completion of the study, we will disseminate the outcomes of the study in a peer-reviewed academic journal. When the training is finalized, the workbooks and manuals will be freely distributed online for CBT therapists.

## Discussion

CBT is an effective method for youth with depressive symptoms [17, 18]. However, a part of adolescents do not seem to benefit from CBT. In the current study, we have tried to optimize and personalize an existing CBT training for adolescents with depressive symptoms by using a modular approach and by making it inclusive of diverse educational levels, cultural backgrounds, and gender. These adjustments will possibly increase adherence and satisfaction, which in turn can increase effectiveness. This simplified and adapted modular CBT training (STARr2.0) for youth with depressive symptoms will be examined. More specifically, we will examine to what extent this adapted modular CBT training for youth with depressive symptoms is effective in reducing depressive symptoms and comorbid stress and anxiety symptoms. In addition, regarding the sequence of modules, we will assess whether the preference of the adolescent, the preference of the trainer, or a data-driven prediction best predicts treatment outcomes. Last, we will examine moderators (gender and age) and mediators (problem-solving approaches, cognitive thinking errors, behavioral activation, stress).

An n-of-1 design, more specifically a SCOD, will be used for this study. Single case studies are a well-fitting design for clinical studies and can indicate the efficacy of CBT [52, 53]. Moreover, n-of-1 designs can identify individuals’ characteristics that might affect the outcome and provide insight into what mechanisms of change contribute to decreasing depressive symptoms. Insight into moderators and mediators can enhance understanding of what works best for whom in CBT [47], which is not yet fully understood.

This study faces several challenges. First, for the inclusion of participants, we are dependent on the registrations of participants at the organizations. We are only able to ask youth to participate in the study after they have registered for the training at one of the two organizations. Further, a minimum of 80% of completed questionnaires from 30 participants is needed to have sufficient power for all planned analyses, while we are aware that dropout from CBT or studies into CBT is prevalent [98, 99]. To reduce drop-out, youth are motivated to complete questionnaires by compensating them with 90 euros when they complete all questionnaires. In addition, there is a strong collaboration between researchers and trainers, so when participants do not complete the questionnaires, trainers can send out reminders immediately. Also, trainers are instructed to motivate participants to complete questionnaires during the training. A last challenge relates to incorporating personalization in group training. Because participants may have diverse preferences and needs, individual training seems a better fit. For example, regarding the sequence of modules, not all participants will receive their preferred sequence of modules. Nevertheless, the STARr training is adapted in such a way that we expect it will meet the needs of a broad group of adolescents regarding educational level, cultural background, sexual orientation, and gender. Also, we want to mimic clinical practice as much as possible. Preventative interventions are often offered in group format to reduce costs. Therefore, we decided to offer the training in a group format and using the majority of preferences to determine the most appropriate sequence.

This study also has several strengths. We will evaluate the adapted STARr intervention in existing mental health care settings among youth who searched for mental care themselves or together with their parents. Participants will not be motivated by the compensation to enroll in the training because they first register to enroll in the STARr training and then they will be informed about the study. This might reflect best the group of youth who would generally enroll in the training, which enhances the external validity. Moreover, we use an innovative design in which we use input of the youth, trainers, and data to predict the best fit of modules for each participant. In addition, by using a single case design, participants are their own control condition, which allows all participants in the study to receive the care they need. Additionally, this design allows us to assess the change mechanisms that underlie the change in depressive symptoms, providing insight into what works for whom and why.

This study will provide insight into whether the adapted STARr training is effective in reducing depressive symptoms and co-occurring anxiety and stress symptoms. Moreover, it will provide insight into moderators and mediators. When the adapted STARr training is effective in reducing depressive symptoms and co-occurring stress and anxiety, the training can be provided widely to youth with elevated levels of depressive symptoms by trained mental health professionals in the Netherlands.

## Data Availability

No datasets were generated or analysed during the current study.

## Abbreviations

BADS: Behavioral Activation for Depression Scale
BPM: Brief Problem Monitor
CBT: Cognitive behavioral therapy
CDI-2: Children’s Depression Inventory-2
CNCEQ-R: Children’s Negative Cognitive Errors Questionnaire – Revised
ESM: Experience sampling method
GDPR: General Data Protection Regulation
ISIQ: Individual and Societal Impact Questionnaire
MYTS: Motivation for Youth’s Treatment Scale
PDT: Permutation distancing test
PETS: Parent Expectancies for Therapy Scale
SCARED: Screen for Child Anxiety Related Emotional Disorders
SCOD: Single case observational design
SPSI-R: Social Problem Solving Inventory-Revised
SSS: Service Satisfaction Scale
STARr: Solve, Think, Act, Relax, repeat
TASC-R: Therapy Alliance Scale for Children-revised
VOZZ: Suicide and self-harm questionnaire
